# Machine-learning-enhanced image reconstruction in optical tomography using the Monte Carlo method for light transport

**DOI:** 10.1117/1.JBO.31.9.096001

**Published:** 2026-09-01

**Authors:** Jonna Kangasniemi, Meghdoot Mozumder, Andreas Hauptmann, Tanja Tarvainen

**Affiliations:** a University of Eastern Finland, Department of Technical Physics, Kuopio, Finland; b University of Oulu, Research Unit of Mathematical Sciences, Oulu, Finland; c University College London, Department of Computer Science, London, United Kingdom

**Keywords:** optical tomography, radiative transfer equation, Monte Carlo method for light transport, machine learning, stochastic optimization

## Abstract

**Significance:**

The Monte Carlo method for light transport is widely accepted as an accurate method for simulating light propagation in a scattering medium. Its use in optical tomography, however, suffers from inherent stochastic noise. This noise is present in both evaluations of the forward model, as well as in the search direction of the minimization algorithm used for image reconstruction.

**Aim:**

We aim to utilize machine learning to compensate for the stochastic Monte Carlo noise in the reconstruction of absorption and scattering in optical tomography.

**Approach:**

An iterative image reconstruction algorithm is proposed. The algorithm uses convolutional neural networks in a stochastic Gauss–Newton update when estimating absorption and scattering coefficients.

**Results:**

The methodology is evaluated using numerical simulations and compared against the conventional stochastic Gauss–Newton algorithm in optical tomography. It is demonstrated that the methodology can be used to compensate for image reconstruction artifacts caused by the stochastic noise.

**Conclusions:**

The proposed machine learning approach can be used to compensate for stochastic noise in Gauss–Newton iterations, and it enables reconstruction of absorption and scattering with a significantly lower number of photons than a conventional stochastic Gauss–Newton algorithm.

## 1 Introduction

Optical tomography (OT) is a biomedical imaging modality where optical parameters of an imaged target are reconstructed from boundary measurements of visible red or near-infrared light.^1^^,^^2^ This noninvasive method can provide unique functional and structural information of biological targets with applications, for example, in functional brain imaging,^3^^,^^4^ breast cancer imaging,^5^ thyroid cancer imaging,^6^ diagnosis of rheumatoid arthritis,^7^^,^^8^ treatment monitoring,^9^ and small animal studies.^10^

Reconstruction of optical parameters in OT is a highly ill-posed inverse problem, that is, even small errors in measurement or modeling can lead to significant errors in the reconstructed images.^1^^,^^11^ The inverse problem in OT is typically formed either as a difference imaging problem or an absolute imaging problem.^1^ In difference imaging, two sets of measurements are used to reconstruct images of the changes in optical parameters between them. In absolute imaging, a single set of measurements is used to reconstruct images of the (absolute) values of the optical parameters. In this work, we consider the absolute reconstruction of absorption and scattering coefficients in OT.

Computational image reconstruction in OT requires modeling of light propagation in the imaging domain during an image reconstruction algorithm. In biological tissues, light propagation can be modeled using the radiative transfer equation (RTE). Generally, in OT, the RTE is approximated using its diffusion approximation. However, when imaging small targets, with a size of less than few scattering lengths, or targets with low-scattering regions, the approximation of diffuse light is not valid, and light propagation needs to be modeled as radiative transfer.^1^^,^^9^ In this work, the solution of the RTE is approximated using the Monte Carlo method for light transport. In the Monte Carlo method, light propagation is approximated by simulating photons (or photon packets) and tracing their trajectories in a medium.^12^ The method is generally accepted as an accurate approach for simulating light propagation in biological tissues. For more information on the Monte Carlo method for light transport, see, e.g., Refs. 13–17 and the references therein.

In this work, the image reconstruction problem of OT is formulated as a minimization problem, where the Monte Carlo method is used as a forward model. Previously in OT, Monte Carlo has been utilized in difference imaging, e.g., in Refs. 18–24. Now, absolute imaging is considered, and the image reconstruction problem is approached following our previously proposed stochastic Gauss–Newton method.^25^^,^^26^ In the approach, the Jacobian matrix for absorption is formed by differentiating the formula for a photon packet weight.^18^^,^^25^^,^^27^ Furthermore, a so-called perturbation approximation for the Monte Carlo method is utilized when constructing the Jacobian matrix for scattering.^25^^,^^26^^,^^28^^,^^29^ As it has been noticed,^25^^,^^26^ the number of photon packets needs to be sufficiently large for the stochastic Gauss–Newton algorithm to converge to the correct minimum. The amount of stochastic noise can be controlled by adjusting the number of photon packets used in the simulations but can still result in computationally expensive simulations.^25^^,^^26^^,^^30^

In this work, we propose utilizing machine learning for correcting the search direction of the stochastic Gauss–Newton algorithm. Previously in OT, machine learning has been utilized in fully learned algorithms,^31^^–^^35^ where a nonlinear mapping is learned from boundary data to the spatially distributed optical parameters, in image post-processing,^36^ where machine learning is used to enhance the image quality after an initial reconstruction, and in combining machine learning with model-based reconstruction methods.^37^^–^^41^ In these previous studies, difference imaging has been used to reconstruct the absorption coefficient,^33^^,^^36^^,^^37^^,^^39^^,^^40^ or both absorption and scattering coefficients.^34^^,^^38^ In Ref. 32, bulk absolute values of absorption and scattering were estimated. Furthermore, absolute imaging of absorption was studied in Refs. 31, 35, and 41. In all of the studies, the diffusion approximation of RTE was used as the model for light propagation. In this work, Monte Carlo method is used. Basically, the proposed approach could be interpreted as a methodology for denoising absorption and scattering estimates; similarly, as in Ref. 42, machine learning was used to denoise photon density images from stochastic Monte Carlo noise.

In this work, an iterative model-based deep learning technique,^43^^–^^46^ sometimes called as an algorithm unrolling, is used to enhance the stochastic Gauss–Newton method when the Monte Carlo method is used as a forward model. Previously, in OT, a similar methodology has been utilized in a Gauss–Newton algorithm to reconstruct 3D absolute absorption and scattering distributions utilizing the diffusion approximation under model uncertainties.^43^ A similar approach was also utilized in Ref. 41, where an iterative Levenberg–Marquardt algorithm with a deep learning component was used to reconstruct the absorption coefficient. We note that the key contribution of this work is the incorporation of deep learning in the stochastic Gauss–Newton reconstruction to mitigate Monte Carlo noise, rather than the development of a new neural-network architecture.

The remainder of the paper is organized as follows. In Sec. 2, OT image reconstruction problem and the Monte Carlo method for light transport, as well as the stochastic Gauss–Newton method, are presented. Furthermore, the proposed machine-learning-enhanced stochastic Gauss–Newton method is introduced. In Sec. 3, numerical simulations are presented, and their results are followed by discussion and conclusions in Sec. 4.

## 2 Methods

In this work, frequency-domain OT, where intensity-modulated light is guided to the imaged target and amplitude and phase delays of transmitted signals are measured, is studied.^2^ The image reconstruction can be formulated as a minimization problem of the form,

$$\underset{x}{\arg\;\min}\{\frac{1}{2}{\|L_{e}(Y_{meas}-Y(x)-\eta_{e})\|}^{2}+\frac{1}{2}{\|L_{x}(x-\eta_{x})\|}^{2}\},$$ (1)

where $Y_{meas}=(Y_{meas,1},\ldots ,Y_{\mathrm{meas},M})\in R^{M}$ is a measurement vector, $Y:R^{2K}\to R^{M}$ is a discretized forward model, and $x={\left(\mu_{a},\mu_{s}\right)}^{T}$ denotes the unknown optical parameters absorption $\mu_{a}=(\mu_{a,1},\ldots ,\mu_{a,K})\in R^{K}$ and scattering $\mu_{s}=(\mu_{s,1},\ldots ,\mu_{s,K})\in R^{K}$. Furthermore, $\eta_{e}$ and $L_{e}$ are the expected value and Cholesky decompositions of the inverse of the covariance matrix of the noise, where $\Gamma_{e}^{-1}=L_{e}^{T}L_{e}$, and $\eta_{x}$ and $L_{x}$ are the expected value and Cholesky decomposition of the inverse of the covariance matrix of the prior, where $\Gamma_{x}^{-1}=L_{x}^{T}L_{x}$.

In this work, numerical evaluation of the forward model Y(x) is based on the Monte Carlo method for light transport. Monte Carlo method simulates the solution of the radiative transfer equation (RTE), which in the frequency domain is of the form,^1^^,^^47^^,^^48^

{(iωc+s^·∇+μs(r)+μa(r))ϕ(r,s^)=μs(r)∫Sd−1Θ(s^·s^′)ϕ(r,s^′)ds^′,r∈Ωϕ(r,s^)={ϕ0(r,s^),r∈∪jϵj,s^·n^<00,r∈∂Ω\∪jϵj,s^·n^<0, (2)

where $\Omega \subset R^{d}$ is a domain with a boundary ∂Ω, and dimensions d=2 or 3, i is the imaginary unit, ω=2πf is the angular modulation frequency of the input signal, where f is the modulation frequency, and $c=c_{0}/n$ is the speed of light in the medium, where n is a refractive index, and $c_{0}$ is the speed of light in a vacuum. In addition, $\hat{s}\in S^{d-1}$ is a unit vector in the direction of interest, $\mu_{a}(r)$ is the absorption coefficient, $\mu_{s}(r)$ is the scattering coefficient, $\phi (r,\hat{s})$ is the radiance, $\phi_{0}(r,\hat{s})$ is a boundary source at a position $\epsilon_{j}$, $\hat{n}$ is an outward unit normal on the boundary, and Θ(s^·s^′) is the scattering phase function. In OT, the Henyey-Greenstein phase function,^49^

Θ(s^·s^′)={12π1−g21+g2−2g−2s^·s^′d=214π1−g2(1+g2−2g−2s^·s^′)3/2d=3, (3)

where −1<g<1 is a scattering anisotropy parameter, is commonly used. The presented boundary condition assumes that, on the boundary, photons can travel in the inward direction only at the source locations $\epsilon_{j}\subset \partial \Omega$. In OT, the data consist of the exitance Y on the boundary of the domain, defined as

Y(r)=∫Sd−1ϕ(r,s^)(s^·n^)ds^. (4)

### 2.1 Monte Carlo Method for Light Transport

In this work, the photon packet Monte Carlo method is used.^12^ In the photon packet method, packets of photons with an initial weight of $w_{0}$ are simulated, and their paths are traced as they undergo absorption and scattering events in the medium.^12^^,^^14^^,^^27^ Let us consider a probability for a photon absorption as $\mu_{a}ds$ and a probability for a photon scattering as $\mu_{s}ds$ in a small length ds in the propagation direction. The probability of a photon absorption is taken into account by reducing the weight of the photon packet along the trajectory s. When considering the frequency-domain simulations, the weight w(s) is

$$w(s)=w_{0}\;\exp\;(-\int_{0}^{s}\mu_{a}(s^{\prime })+\frac{i\omega }{c}ds^{\prime }).$$ (5)

The path length that the photon travels before the scattering event is described by a scattering length l, which follows an exponential probability density function,

$$f(l)=\mu_{s}(l)\;\exp(-\int_{0}^{l}\mu_{s}(l^{\prime })dl^{\prime }).$$ (6)

The probability density function of the scattering angle, which in this work is the Henyey-Greenstein phase function [Eq. (3)], defines the photon packet direction after a scattering event. During a Monte Carlo simulation, a path of a photon packet is followed until it exits the simulation domain or it is terminated.^12^ Then, this simulation is repeated using new photon packets until an adequate statistics for simulating light propagation is obtained.

The exitance [Eq. (4)] on a boundary element b can be calculated as

$$Y=\frac{W_{b}}{PA_{b}}=\frac{1}{PA_{b}}\underset{\mathrm{phot}}{\sum }w_{\mathrm{phot}}(s)\;=\frac{1}{PA_{b}}\underset{\mathrm{phot}}{\sum }w_{0}\;\exp(-\underset{h}{\sum }\left(\mu_{a,h}+\frac{i\omega }{c}\right)l_{h,\mathrm{phot}}),$$ (7)

where $A_{b}$ is the width (d=2) or area (d=3) of the boundary element b, P is the number of simulated photon packets, $W_{b}$ is the weights of the photon packets that exited the simulation domain through the boundary element b, “phot” denotes the photon packets that escaped through the boundary element b, and $l_{h,\mathrm{phot}}$ is the discretized photon path length in a discretization element h of a piece-wise constant discretized optical parameters.^18^^,^^25^^,^^27^

### 2.2 Stochastic Gauss–Newton Method

Due to the stochastic nature of the Monte Carlo method, the minimization problem [Eq. (1)] is also stochastic, and it can be approached using methods of stochastic optimization,^30^^,^^50^ such as the stochastic Gauss–Newton (SGN) method.^25^^,^^26^^,^^30^ In the SGN method, the optical parameters $x_{i}$ are updated by

$$x_{i+1}=x_{i}+\Delta x_{i},$$ (8)

where $\Delta x_{i}=\alpha_{i}\delta (x_{i})$ is the Gauss–Newton step, $\delta (x_{i})$ is the Gauss–Newton search direction calculated with P photon packets, and $\alpha_{i}$ is the step length parameter, which in this work is set as α=1. The SGN search direction is of the form,

$$\delta (x_{i})={\left(J^{T}(x_{i})\Gamma_{e}^{-1}J(x_{i})+\Gamma_{x}^{-1}\right)}^{-1}(J^{T}(x_{i})\Gamma_{e}^{-1}(Y_{meas}-Y(x_{i})-\eta_{e})-\Gamma_{x}^{-1}(x_{i}-\eta_{x})),$$ (9)

where $Y(x_{i})$ is the stochastic forward model and $J(x_{i})=(J_{\mu_{a}}(x_{i}),J_{\mu_{s}}(x_{i}))$ is the Jacobian matrix of the forward model evaluated with P photon packets at the update $x_{i}$.

To construct the Jacobian matrix, the absorption and scattering derivatives of exitance are needed. The absorption derivative of exitance can be calculated directly from Eqs. (4) and (7),^18^^,^^25^ i.e., on a boundary element b in a discretization element k, the derivative of the exitance $Y_{b}$ for the absorption $\mu_{a,k}$ is

$$\frac{\partial Y_{b}}{\partial \mu_{a,k}}=\frac{1}{PA_{b}}\underset{\mathrm{phot}}{\sum }-l_{k,\mathrm{phot}}w_{\mathrm{phot}}(s)\;=\frac{1}{PA_{b}}\underset{\mathrm{phot}}{\sum }-l_{k,\mathrm{phot}}w_{0}\;\exp(-\underset{h}{\sum }(\mu_{a,h}+\frac{i\omega }{c})l_{h,\mathrm{phot}}).$$ (10)

The scattering derivative of exitance can be calculated utilizing a perturbation Monte Carlo method,^27^^,^^28^^,^^51^ in which the effect of a perturbation in the optical parameters is evaluated by reusing the trajectories from unperturbed simulations.^19^^,^^29^ The perturbed weight $\overset{˜}{w}$ of a perturbed scattering coefficient ${\overset{˜}{\mu }}_{s}$ can be obtained by

$$\overset{˜}{w}=w{\left(\frac{{\overset{˜}{\mu }}_{s}}{\mu_{s}}\right)}^{n_{s}}\;\exp\;\left(-\left({\overset{˜}{\mu }}_{s}-\mu_{s}\right)L_{\mathrm{tot}}\right),$$ (11)

where w is the unperturbed weight, $n_{s}$ is the number of scattering events in the perturbed region, and $L_{\mathrm{tot}}$ is the total distance that the photon packet has traveled inside the perturbed region.^19^^,^^29^ Using the perturbation approximation, the derivative of exitance $Y_{b}$ with respect to scattering coefficient $\mu_{s,k}$ on boundary element b in a discretization element k is^25^

$$\frac{\partial Y_{b}}{\partial \mu_{s,k}}=\frac{1}{PA_{b}}\underset{\mathrm{phot}}{\sum }(\frac{n_{s}}{\mu_{s,k}}-l_{k,\mathrm{phot}})w_{\mathrm{phot}}(s)\;=\frac{1}{PA_{b}}\underset{\mathrm{phot}}{\sum }(\frac{n_{s}}{\mu_{s,k}}-l_{k,\mathrm{phot}})w_{0}\;\exp\left(-\underset{h}{\sum }(\mu_{a,h}+\frac{i\omega }{c})l_{h,\mathrm{phot}}\right).$$ (12)

### 2.3 Deep Stochastic Gauss–Newton Method

In this work, we propose an approach for improving the stochastic Gauss–Newton update, Eq. (8), utilizing a machine learning approach.^43^^–^^46^ In the proposed approach, the stochastic Gauss–Newton update [Eq. (8)] is replaced with a learned update function,

$$x_{i+1}=G_{\theta_{i}}(x_{i},\Delta x_{i}),$$ (13)

where the functions $G_{\theta_{i}}$ correspond to convolutional neural networks (CNNs) with a chosen architecture and different learned parameters on each iteration i. Thus, we can write a deep stochastic Gauss–Newton (DSGN) algorithm, where the (noisy) update of the optical parameters $\Delta x_{i}$ is corrected on each iteration using a CNN.^43^^,^^44^

**Fig. 1:**
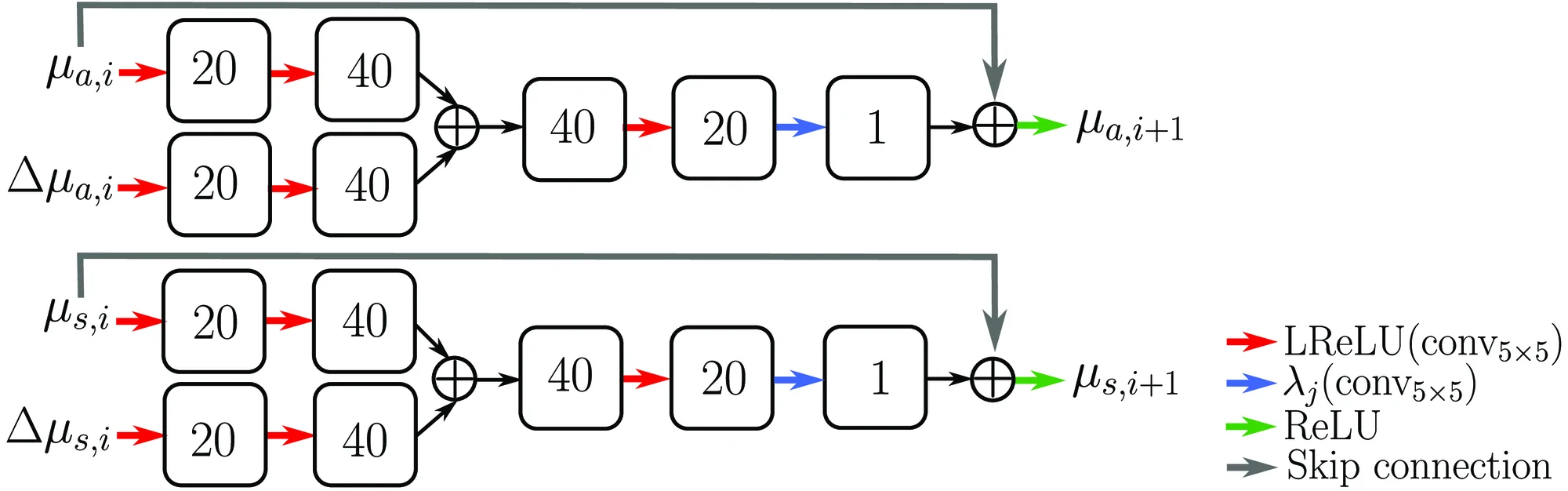
Illustration of one convolutional neural network denoted as $G_{\theta_{i}}$, representing one deep stochastic Gauss–Newton iteration. A convolutional layer with a 5×5 kernel, followed by a LReLU, is marked with red arrows. A convolutional layer with a 5×5 kernel, followed by a scalar multiplication $\lambda_{j}$, is marked with a blue arrow. The number of resulting channels is marked inside the squares. With the skip connection (gray arrow), the residual update is projected to the positive numbers by the last ReLU, marked with a green arrow.

The algorithm for the training of DSGN is shown in Algorithm 1. To take into account the nonlinearity of the OT image reconstruction problem, an iterative unrolled method is considered.^44^ This means that a CNN is applied layer by layer to the optical parameters $x_{i}=(\mu_{a,i},\mu_{s,i}{)}^{T}$ and the stochastic Gauss–Newton update $\Delta x_{i}=(\Delta \mu_{a,i},\Delta \mu_{s,i}{)}^{T}$ on each iteration. During the training phase, on each iteration, the true absorption and scattering distributions, the current Gauss–Newton estimates $x_{i}$, and the current Gauss–Newton update $\Delta (x_{i})$ calculated with P photon packets are provided as input to the pipeline. To improve numerical stability, both absorption and scattering are reparametrized to have values in the same range using a change of variables.^52^ The training data consist of true absorption and scattering images $\{\mu_{a,\mathrm{true}},\mu_{s,\mathrm{true}}{\}}^{j},j=1,\ldots ,N_{\mathrm{samp}}$, where $N_{\mathrm{samp}}$ is the number of samples. These samples are used to simulate OT data and SGN updates using the Monte Carlo method, and then, they are corrupted with additive noise. In this work, the network parameters $\theta_{i}$ for i=1,…,I, where I is the number of iterations, are trained sequentially by minimizing the $L_{2}$-loss function for all samples j, given by

$$\underset{\theta_{i}}{\min}\underset{j}{\sum }\|\mu_{a,i+1}^{j}-\mu_{a,\mathrm{true}}^{j}{\|}^{2}+\|\mu_{s,i+1}^{j}-\mu_{s,\mathrm{true}}^{j}{\|}^{2},$$ (14)

where the updates $\mu_{a,i+1}^{j}$ and $\mu_{s,i+1}^{j}$ are given in Eq. (13). The architecture of the CNNs used in this work is based on a previous study,^44^ and it is shown in Fig. 1. Variants of this architecture have also been earlier used in other tomographic applications, see, e.g. Refs. 43, 53, and 54. The architecture consists of a feature-extraction part and a reassembling part. In the network, absorption and scattering parameters are handled in separate channels. First, the parameter maps are expanded to 20 and then 40 channels by a convolutional layer with a 5×5 kernel and dimension d=2. A “leaky” rectified linear unit (LReLU) is used as an activation function to allow negative values for the input parameters. In the reassembling part, the output of these two pipelines is added together and reduced first to 20 channels with LReLU as an activation function and then to 1 channel with a scalar multiplication. Finally, the result is added to the parameter values at the current iterate and projected to the positive numbers using a rectified linear unit (ReLU) as an activation function. The architecture was kept simple as the main contribution of this work was not to propose a specific CNN architecture.

**Algorithm 1:** Training the Deep Stochastic Gauss–Newton

1: Draw set of $\{x_{\mathrm{true}}{\}}^{j}$, $j=1,\ldots ,N_{\mathrm{samp}}$, from a prior model.
2: Simulate data with the Monte Carlo method (Sec. 2.1), and corrupt it with additive noise.
3: Set $x_{1}^{j}$ to a chosen initial value ∀j, for example, the mean of the prior.
4: Set i←1.
5: **while** i<I **do**
6: Compute ${\left(\delta x_{i}\right)}^{j}$ with P photon packets ∀j using [Eq. (9)], and set $\alpha_{i}$.
7: **function** TRAIN($\left(x_{i}^{j},{\left(\Delta x_{i}\right)}^{j},x_{\mathrm{true}}^{j}\right)$, ∀j)
8: Train parameters $\theta_{i}$ by minimizing [Eq. (14)].
9: **end function** Return $\theta_{i}$
10: Set $x_{i+1}^{j}\leftarrow G_{\theta_{i}}(x_{i}^{j},{\left(\Delta x_{i}\right)}^{j})$, ∀j.
11: Set i←i+1
12: **end while**

The DSGN algorithm can be evaluated by applying the learned network $G_{\theta_{i}}$ with the trained parameter set $\theta_{i}$ to the optical parameters and Gauss–Newton update on each iteration i. The evaluation corresponds to Algorithm 1, starting by setting $x_{1}^{j}$ and skipping the “TRAIN” function.

## 3 Simulations

Two-dimensional numerical simulations were used to evaluate the proposed methodology. The minimization algorithm and simulations were implemented in MATLAB (R2024a, MathWorks Inc., Natick, Massachusetts, United States). In the implementation and training of the deep stochastic Gauss–Newton, the Python library TensorFlow (version 2.18) was utilized. The Monte Carlo simulations were implemented in C++. The simulations were carried out in an AMD Ryzen Threadripper PRO 7965WX 24-core CPU compute server with 48 logical threads and 250 GiB RAM. The training of the DSGN was performed on an NVIDIA RTX 6000 Ada Generation GPU with 49,140 MiB of memory.

### 3.1 Simulation Geometries and Discretization

The simulation domain was a 5  mm×5  mm square. In the simulation setup, 20 sources (five on each side) and 100 detectors (25 on each side) with a width of 0.2 mm were considered. The sources were placed on each side with a separation of 0.5 mm between two sources, centrally such that the distance from the outermost sources was 1 mm to the corner of the square. The detectors were placed side to side, covering the side length of the simulation domain. One source was used at a time, and data were recorded on detectors on the adjacent and opposite sides of the square.

**Fig. 2:**
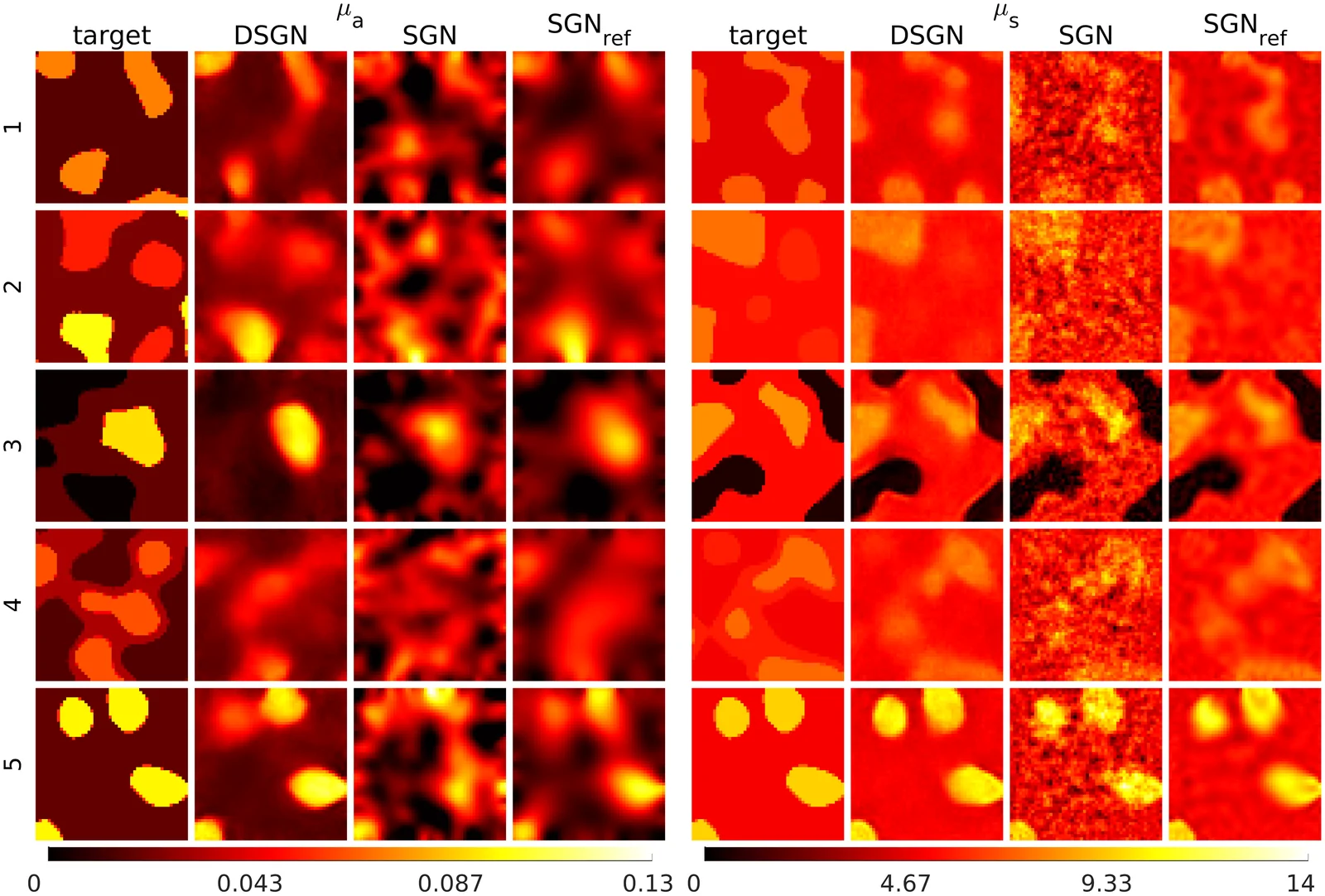
Reconstructed absorption coefficients $\mu_{a}\;\left({\mathrm{mm}}^{-1}\right)$ (columns 1 to 4) and scattering coefficient $\mu_{s}\;\left({\mathrm{mm}}^{-1}\right)$ (columns 5 to 8) of test targets corresponding to the training set. The true targets are shown in columns 1 and 5, deep stochastic Gauss–Newton reconstructions (DSGN) in columns 2 and 6, stochastic Gauss–Newton reconstructions using $10^{6}$ photon packets (SGN) in columns 3 and 7, and stochastic Gauss–Newton reconstruction using $10^{9}$ photon packets (${\mathrm{SGN}}_{\mathrm{ref}}$) in columns 4 and 8.

For data simulation, the absorption and scattering, and the photon density were expressed in a piecewise constant triangular mesh with 5000 elements for training and validation. For the testing data, a triangular mesh with 5000 elements was used for the in-distribution targets, which are shown in the first column in Fig. 2. For out-of-distribution targets, shown later in Fig. 4, 9006 elements were used for the circle target and 20,000 elements for the vessel target. In image reconstruction and training, absorption and scattering were presented in a piecewise constant discretization of 50×50 pixels grid, i.e., 2500 pixels, and the photon density was simulated in a triangular mesh consisting of 5000 elements.

### 3.2 Data Simulation

The optical parameters of the targets were chosen to mimic the optical properties of a biological tissue.^55^^,^^56^ In all simulations, the refractive index was n=1, the scattering anisotropy parameter was g=0.9, and the frequency of the intensity-modulated light was f=100  MHz. In addition, the light source shape was modeled as angularly cosine and spatially uniform in the Monte Carlo implementation.

The training data, the validation targets, and the in-(training)-distribution test targets were simulated by segmenting images of optical parameters drawn from a squared exponential prior,^57^ using K-means. Then, the absorption and scattering values for the backgrounds and inclusions were drawn from uniform distributions. Examples of these absorption and scattering images are shown in Fig. 2. In total, 800 training targets and 200 validation targets were simulated. In addition, five in-distribution test targets and two out-of-distribution targets (Fig. 4), i.e., a circle target and a vessel target, were considered. The vessel target was modified from a numerical breast phantom.^58^

The photon density and exitance were simulated with the Monte Carlo method, as described in Sec. 2.1. The data were simulated using $10^{8}$ photon packets for each source. As a data type, the amplitude and phase of the complex exitance [Eq. (7)] were considered. The simulated noiseless data were corrupted with additive, Gaussian-distributed random noise with zero mean and standard deviation that corresponds to 0.5% of the relative magnitude of the noiseless data to simulate realistic, i.e., high enough, noise.

### 3.3 Training

The network was trained as described in Sec. 2.3 using absorption and scattering images of the training set and the corresponding Monte Carlo simulated data. During the training and validation, the update directions for DSGN [Eq. (9)] were calculated using $10^{6}$ photon packets. As an initial guess $\mu_{1}^{j}$, a scaled mean of the prior was used. The parameters $\theta_{i}$ were trained by minimizing [Eq. (14)] using TensorFlow’s implementation of Adam optimizer^59^ using batch size 8, 25 epochs, and learning rate $5\cdot 10^{4}$. The number of iterations I was set to 20, which, according to our simulations, provided convergence of the stochastic Gauss–Newton iterations in this simulation setup. The same trained network was used in all DSGN reconstruction studies in this work.

### 3.4 Reconstructions

The DSGN was used to approximate the solution of the minimization problem [Eq. (1)] defined by the loss function [Eq. (14)] (Sec. 2.3). The results were compared with the solution solved using the SGN method (Sec. 2.2). In the image reconstruction, the noise was modeled uncorrelated Gaussian noise with zero mean, and standard deviation $\sigma_{e}$ was specified as 0.5% of the relative magnitude of the simulated noisy data. Furthermore, the Ornstein–Uhlenbeck model was used as a prior model.^57^ The Ornstein–Uhlenbeck prior belongs to the class of Matèrn covariance functions, and based on the authors’ previous studies, it is an effective and flexible prior model for different imaged targets. The covariance matrix of the Ornstein–Uhlenbeck prior is defined as,

$$\Gamma_{x}(i,j)=\sigma_{x}^{2}\;\exp\;(-\left\|r_{i}-r_{j}\right\|/\tau ),$$ (15)

where $\sigma_{x}$ is the standard deviation, τ denotes the characteristic length scale parameter controlling the spatial smoothness, and $r_{i}$ and $r_{j}$ are discretization points. In all simulations, the prior mean for absorption was $\eta_{\mu_{a}}=0.02\;{\mathrm{mm}}^{-1}$ and for scattering $\eta_{\mu_{s}}=5\;{\mathrm{mm}}^{-1}$. The standard deviation for absorption was $\sigma_{\mu_{a}}=0.0267\;{\mathrm{mm}}^{-1}$ and for scattering was $\sigma_{\mu_{s}}=1.67\;{\mathrm{mm}}^{-1}$. The standard deviation values were set so that the maximum possible contrast in absorption and scattering corresponded to three standard deviations from the background. The characteristic length scale was τ=0.5  mm.

To improve the numerical stability of the simultaneous reconstruction of absorption and scattering, the optical parameters were reparametrized to the same range by a division of the prior means.^52^ The scaled prior mean was used as an initial value of the Gauss–Newton for all simulations. In the DSGN method, on each iteration, $10^{6}$ photon packets were simulated on each source. In the SGN method, the reconstructions were evaluated using $10^{6}$ and $10^{9}$ photon packets on each source for each iteration. In all reconstructions, the number of iterations was 20, which was chosen to provide comparable results between all reconstructions. It should be noted that, when applying the methodology in practice, one would choose a convergence criterion to end the iteration immediately after the minimization problem has converged, see, e.g. Ref. 60.

### 3.5 Results

Reconstructed absolute absorption and scattering images for five in-distribution test targets are shown in Fig. 2. As can be seen, the DSGN method provides reconstructions that highly resemble the reference reconstructions calculated with SGN using $10^{9}$ photon packets. The locations of the inclusions can be distinguished using both DSGN and SGN. Furthermore, the contrast of the scattering images is similar when compared with the true targets. In the absorption reconstructions, the contrast of the DSGN method is slightly better than the contrast of the SGN reconstructions with a high number of photon packets when compared with the true targets. When comparing the DSGN reconstruction to the SGN using the same number of photon packets ($10^{6}$), the reconstructions are less noisy, and the inclusions are better distinguished with DSGN, especially when comparing the scattering reconstructions. In all of the reconstructed images, the background is smoother in the DSGN reconstruction than in the SGN reconstructions.

**Fig. 3:**
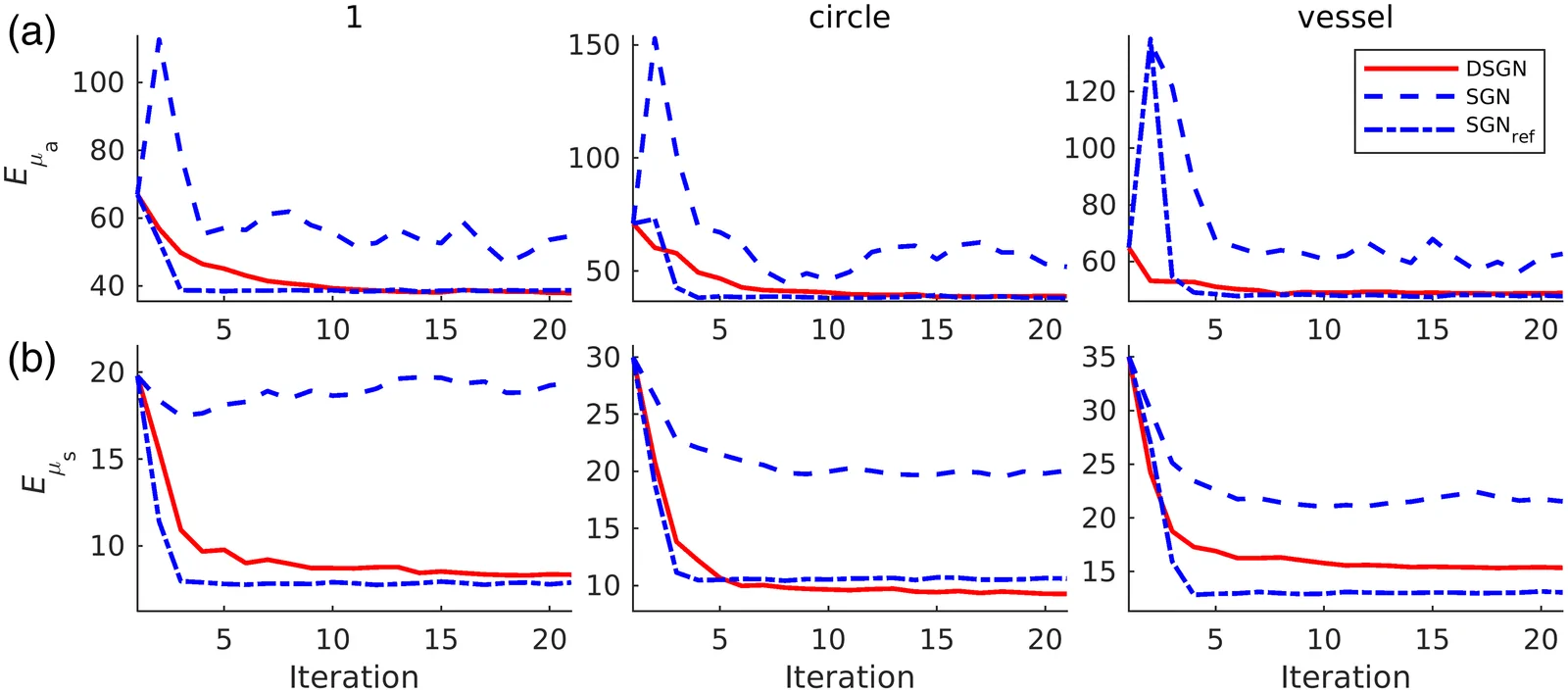
Relative error of absorption $E_{\mu_{a}}(\%)$ (a) and scattering $E_{\mu_{s}}(\%)$ (b) against the iteration. Columns from left to right: the relative errors of of the reconstructions of the target in the first row of Fig. 2 (first column), the circle target (second column), and the vessel target (third column), reconstructed using the deep stochastic Gauss–Newton method (DSGN, solid red line), and the stochastic Gauss–Newton method with $10^{6}$ photon packets (SGN, dashed blue line) and $10^{9}$ photon packets (${\mathrm{SGN}}_{\mathrm{ref}}$, dash-dotted blue line).

In addition to visual comparison, the results were compared by calculating the relative errors for absorption and scattering,

$$E_{\mu_{a}}=\frac{\left\|\mu_{a}-\mu_{a,\mathrm{true}}\right\|}{\left\|\mu_{a,\mathrm{true}}\right\|}\cdot 100\%,\;E_{\mu_{s}}=\frac{\left\|\mu_{s}-\mu_{s,\mathrm{true}}\right\|}{\left\|\mu_{s,\mathrm{true}}\right\|}\cdot 100,$$ (16)

where the norm is the Euclidean norm, and $\mu_{a}$ and $\mu_{s}$ are the reconstructed and $\mu_{a,\mathrm{true}}$ and $\mu_{s,\mathrm{true}}$ are the true absorption and scattering coefficients interpolated to the reconstruction discretization. The relative errors against the iteration for the absorption and scattering reconstructions of the target in the first row of Fig. 2 are shown in the first column of Fig. 3. Based on the relative errors, the DSGN converges to the same level as the SGN with a high number of photon packets. In practice, the SGN with a high number of photon packets converged in six iterations, and the DSGN converged in 16 iterations when a convergence criterion where the relative difference between the estimates of three consecutive iterations is less than 5% was applied.^60^ The SGN with $10^{6}$ photon packets suffers from higher stochastic noise, and it did not converge with the same criterion as other iterations. Notice also that in SGN reconstruction with $10^{6}$ photon packets, the relative error of the absorption gets higher values than the initial guess on the first iteration. We believe that it is caused by the cross-talk effect between absorption and scattering estimates, and the DSGN can correct for this effect.^43^

The computation times for forward (data) simulation, training, and reconstructions using the DSGN and SGN approaches were recorded for the target in Fig. 2(a). The data simulation using $10^{8}$ photon packets took 321 s. The times for forward and Jacobian evaluation, running the Python training/evaluation script from MATLAB, neural-network inference, one Gauss–Newton iteration, and total computation time of training for 20 iterations, and reconstruction algorithm until convergence are given in Table 1. The $t_{\mathrm{python}}$ corresponds to the total time required to execute the Python training (or evaluation) script from MATLAB using the MATLAB built-in function system. This includes the time required to launch Python, to import the needed Python libraries, e.g., TensorFlow, and the network computation time $t_{\mathrm{network}}$. As can be seen, when training the DSGN, the most time-consuming part is the forward and Jacobian evaluation for the 800 training and 200 validation targets, which takes approximately 9600 s in total for one DSGN iteration. In addition, running the Python training script takes approximately 75 s, while the training itself takes approximately 15 s for one iteration. The DSGN for one iteration is around 160 times faster than the SGN iteration with $10^{9}$ photon packets. Because the DSGN converges more slowly (16 iterations) than the SGN reference reconstruction (6 iterations), the total computation time of the DSGN is around 60 times faster than the reference SGN. On the other hand, when comparing the DSGN iteration time to the SGN with the same number of photon packets ($10^{6}$), the DSGN is approximately three times slower than the SGN.

**Table 1:** Computation times of training, deep stochastic Gauss–Newton (DSGN), and stochastic Gauss–Newton (SGN) algorithms using P photon packets for the target in the first row of Fig. 2. Time for forward and Jacobian evaluation ($t_{Y,J}$), running the Python training/evaluation script from MATLAB ($t_{\mathrm{python}}$), network computation ($t_{\text{net}\mathrm{work}}$), one Gauss–Newton iteration ($t_{\mathrm{iter}}$) averaged over 20 runs, and total computation time of training for 20 iterations and reconstruction algorithm until convergence $t_{\mathrm{tot}}$. SGN algorithm using $10^{6}$ photon packets did not converge to the desired criteria.

	P	$t_{Y,J}(s)$	$t_{\mathrm{python}}(s)$	$t_{\mathrm{network}}(s)$	$t_{\mathrm{iter}}(s)$	$t_{\mathrm{tot}}(s)$
Training	$10^{6}$	9	75	15	9,613	192,260
DSGN	$10^{6}$	9	18	1	28	448
SGN	$10^{6}$	9	—	—	10	—
$SGN_{ref}$	$10^{9}$	4,546	—	—	4,547	27,282

Due to the stochasticity of the SGN method, the result may vary when a different set of photon packet realizations is used in image reconstruction. To provide statistical information on the performance of the methods, DSGN and SGN reconstructions calculated using $10^{6}$ photon packets were repeated 100 times. Then, the mean and standard deviations of the relative error of the absorption $E_{\mu_{a}}$ and the scattering $E_{\mu_{s}}$ and the structural similarity index of the absorption ${\mathrm{SSIM}}_{\mu_{a}}$ and the scattering ${\mathrm{SSIM}}_{\mu_{s}}$ were calculated. The relative errors of the SGN reconstruction obtained with $10^{9}$ photon packets were considered a reference. The reference values and the mean and standard deviations of the relative errors and structural similarity indices are presented in Table 2. Furthermore, to study the methodology over a set of targets, the means and standard deviations of relative errors and structural similarity indices were calculated for 100 absorption and scattering reconstructions from data of different in-distribution targets. These are also given in Table 2. As can be seen, the DSGN method provides lower relative errors and higher SSIM values when compared with the SGN method evaluated with $10^{6}$ photon packets, and similar relative errors and SSIM values when compared with the SGN with $10^{9}$ photon packets. In addition, the standard deviations of the relative errors are smaller when comparing the DSGN with the SGN with $10^{6}$ photon packets. When comparing the standard deviations of the structural similarity index, it can be seen that the standard deviations are smaller with the DSGN absorption reconstructions and the same with the DSGN scattering reconstructions as with the SGN using $10^{6}$ photon packets. It should be noted that the SSIM has been shown to behave poorly with noisy images.^61^ In addition, SSIM is highly sensitive to contrast, which can be seen especially in cases where edges are misaligned.^61^

**Table 2:** Mean and standard deviations of the relative errors $E_{\mu_{a}}(\%)$ and $E_{\mu_{s}}(\%)$, and the structural similarity indexes ${\mathrm{SSIM}}_{\mu_{a}}$ and ${\mathrm{SSIM}}_{\mu_{s}}$ of absorption and scattering estimates calculated using the deep stochastic Gauss–Newton (DSGN) and the stochastic Gauss–Newton (SGN) methods using $10^{6}$ photon packets, and their reference values ($SGN_{ref}$) calculated using the stochastic Gauss–Newton with $10^{9}$ photon packets. Rows 1 to 5: statistics calculated from 100 repeated reconstructions using data of in-distribution test targets 1 to 5 (Fig. 2). Row 6: statistics of reconstructions calculated across of a set of 100 in-distribution test targets.

	DSGN	SGN	${\mathrm{SGN}}_{\mathrm{ref}}$	DSGN	SGN	${\mathrm{SGN}}_{\mathrm{ref}}$
	$E_{\mu_{a}}(\%)$			${\mathrm{SSIM}}_{\mu_{a}}$		
1	37.6±2.3	54.2±3.7	39.2	0.90±0.01	0.75±0.03	0.86
2	34.8±1.9	48.6±3.1	33.8	0.88±0.01	0.78±0.02	0.88
3	35.8±2.3	54.1±3.6	36.2	0.86±0.01	0.74±0.03	0.86
4	39.7±2.7	51.2±5.3	32.7	0.89±0.01	0.80±0.03	0.92
5	35.4±1.6	51.1±3.7	35.9	0.90±0.01	0.74±0.02	0.85
Set	36.9±7.2	52.8±8.5		0.89±0.04	0.76±0.04	
	$E_{\mu_{s}}(\%)$			${\mathrm{SSIM}}_{\mu_{s}}$		
1	8.3±0.2	19.4±0.6	8.2	0.42±0.01	0.20±0.01	0.40
2	4.9±0.2	17.5±0.5	5.8	0.30±0.01	0.08±0.01	0.24
3	12.7±0.4	20.9±0.7	11.7	0.55±0.01	0.38±0.01	0.55
4	5.4±0.2	17.8±0.5	5.7	0.49±0.01	0.14±0.01	0.48
5	8.9±0.4	19.0±0.6	9.9	0.42±0.01	0.22±0.01	0.33
Set	8.9±1.9	18.9±1.6		0.36±0.06	0.19±0.05	

#### 3.5.1 Generalization to out-of-distribution targets

**Fig. 4:**
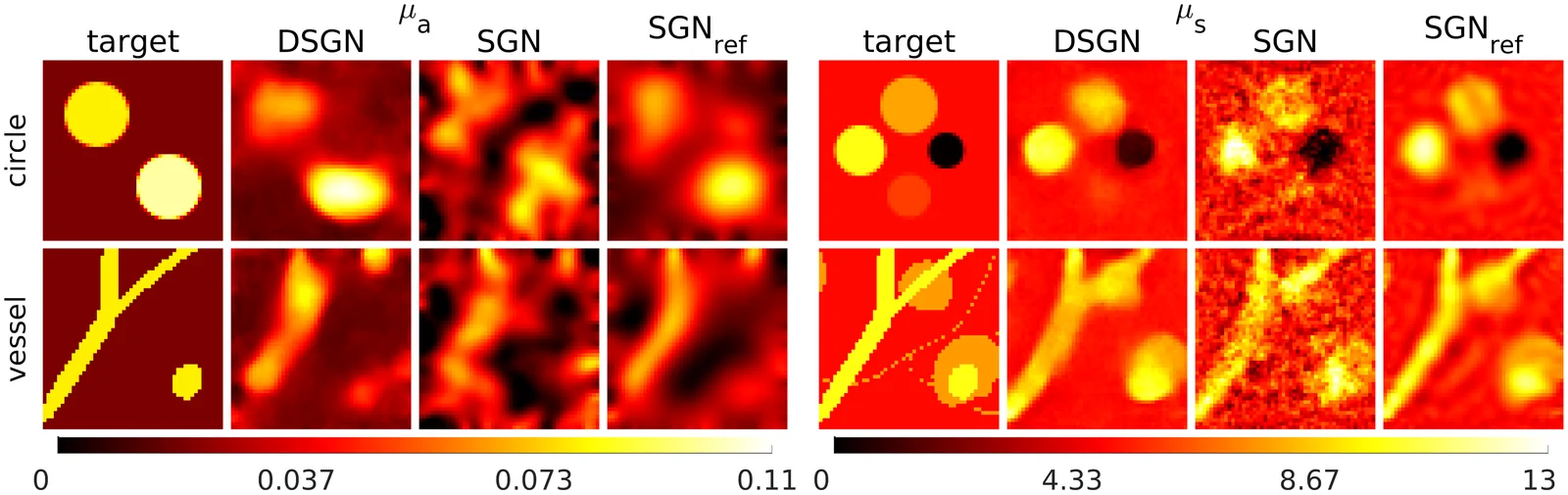
Reconstructed absorption coefficient $\mu_{a}\;({\mathrm{mm}}^{-1})$ (columns 1 to 4) and scattering coefficient $\mu_{s}\;({\mathrm{mm}}^{-1})$ (columns 5 to 8) of the circle and the vessel targets shown in rows 1 and 2, respectively. True targets are shown in columns 1 and 5, deep stochastic Gauss–Newton reconstructions (DSGN) in columns 2 and 6, stochastic Gauss–Newton reconstruction using $10^{6}$ photon packets (SGN) in columns 3 and 7, and stochastic Gauss–Newton reconstruction using $10^{9}$ photon packets (${\mathrm{SGN}}_{\mathrm{ref}}$) in columns 4 and 8.

Generalization of the DSGN method was tested using two out-of-distribution targets, a circle target and a vessel target. The circle target and reconstructions using DSGN and SGN with $10^{6}$ and $10^{9}$ photon packets are shown in the first row of Fig. 4, and the vessel target with the reconstructions is shown in the second row of Fig. 4. It can be seen that the DSGN reconstruction with the circle target provides a reconstruction with a similar quality as the SGN with $10^{9}$ photon packets. On the other hand, when compared with the SGN with the same amount of photon packets, in the DSGN reconstruction, the shape of the inclusions is rounder, and localization is better, especially in the absorption reconstruction. In addition, the contrast of the absorption inclusions is improved using the DSGN. The scattering reconstruction using DSGN is noticeably less noisy than the SGN with the same number of photon packets. In the DSGN reconstruction of the vessel target, on the other hand, the vessel inclusion of the absorption is divided into round shapes. In addition, the vessel in the scattering image is wider and lower in contrast when compared with the SGN estimate with $10^{9}$ photon packets. When comparing the DSGN reconstruction to the SGN with the same amount of photon packets, it can be seen that the shape of the inclusions is better distinguished in both absorption and scattering reconstructions. The DSGN reconstruction converged in 13 iterations with the circle data and in 16 iterations with the vessel data when a convergence criterion where the relative difference between the estimates of three consecutive iterations is less than 5% was applied. The SGN reconstructions with the same number of photon packets did not converge. Furthermore, the SGN with $10^{9}$ photon packets converged in nine iterations with circle data and in eight iterations with vessel data.

The relative errors with respect to the iteration for absorption and scattering estimates are shown in the second column of Fig. 3 for the circle target and in the third column of Fig. 3 for the vessel target. In addition, the mean and the standard deviations of the relative errors and the SSIM, calculated from 100 repeated reconstructions, and their reference values obtained with SGN using $10^{9}$ photon packets, are shown in Table 3. The relative errors and the structural similarity indexes of DSGN with the circle target are at the level of the SGN with $10^{9}$ photon packets. In the reconstruction of the vessel target, the relative errors are slightly higher, and the structural similarity index of the scattering is slightly lower using the DSGN when compared with the SGN with $10^{9}$ photon packets. The structural similarity index of absorption reconstruction with DSGN is at the level when compared with the value of the reference SGN reconstruction. When comparing DSGN with the SGN with the same amount of photon packets, the means of relative errors are lower, and the means of structural similarity indexes are higher, both in absorption and scattering reconstructions. In addition, the standard deviations of the relative errors and structural similarity indexes of DSGN estimates are smaller, except for the SSIM of scattering of the vessel target, where the standard deviation is the same, when comparing to the SGN with $10^{6}$ photon packets.

**Table 3:** Mean and standard deviations of the relative errors $E_{\mu_{a}}(\%)$ and $E_{\mu_{s}}(\%)$, the structural similarity indexes ${\mathrm{SSIM}}_{\mu_{a}}$ and ${\mathrm{SSIM}}_{\mu_{s}}$, position errors $E_{\mu_{a},\mathrm{pos}}$ and $E_{\mu_{s},\mathrm{pos}}$, and spatial overlap $Ov_{\mu_{a}}$ and $Ov_{\mu_{s}}$ of absorption and scattering estimates calculated from 100 repeated reconstructions using the deep stochastic Gauss–Newton (DSGN) and the stochastic Gauss–Newton (DGN) methods using $10^{6}$ photon packets, and their reference values calculated using the stochastic Gauss–Newton method (${\mathrm{SGN}}_{\mathrm{ref}}$) with $10^{9}$ photon packets for the circle and the vessel targets.

	Circle	Vessel	Circle	Vessel	Circle	Circle
	$E_{\mu_{a}}(\%)$		${\mathrm{SSIM}}_{\mu_{a}}$		$E_{\mu_{a},\mathrm{pos}}(\mathrm{mm})$	$Ov_{\mu_{a}}$
DSGN	38.5±2.0	50.0±1.7	0.89±0.01	0.85±0.01	0.031±0.011	0.55±0.07
SGN	53.3±4.7	65.0±4.4	0.74±0.03	0.67±0.04	0.054±0.027	0.27±0.17
${\mathrm{SGN}}_{\mathrm{ref}}$	38.1	47.8	0.87	0.82	0.032	0.46
	$E_{\mu_{s}}(\%)$		${\mathrm{SSIM}}_{\mu_{s}}$		$E_{\mu_{s},\mathrm{pos}}(\mathrm{mm})$	$Ov_{\mu_{s}}$
DSGN	9.5±0.4	15.7±0.3	0.38±0.01	0.38±0.01	0.005±0.002	0.81±0.05
SGN	20.1±0.7	21.3±0.7	0.17±0.01	0.28±0.01	0.007±0.004	0.26±0.09
${\mathrm{SGN}}_{\mathrm{ref}}$	10.6	13.1	0.33	0.45	0.005	0.49

In the case of the circle target, two more error metrics: position error and spatial overlap^62^^,^^63^ were computed to evaluate the localization errors. The position errors for absorption $E_{\mu_{a},\mathrm{pos}}$ and scattering $E_{\mu_{s},\mathrm{pos}}$ were computed as the Euclidean distance between the center of the mass of the reconstructed image and the center of mass of the simulated target image. The spatial overlap for absorption $Ov_{\mu_{a}}$ and scattering $Ov_{\mu_{s}}$ was quantified using the Dice coefficient, defined as the ratio of overlapping voxels between the highest contrast inclusion in the reconstructed and target images. In addition, these were calculated from 100 repeated reconstructions. The metrics are reported in Table 3, where it can be observed that the DSGN and reference SGN reconstructions show comparable position errors and that the DSGN provides improved spatial overlap (Dice coefficients) compared with the SGN reconstructions.

#### 3.5.2 Generalization to 3D data

**Fig. 5:**
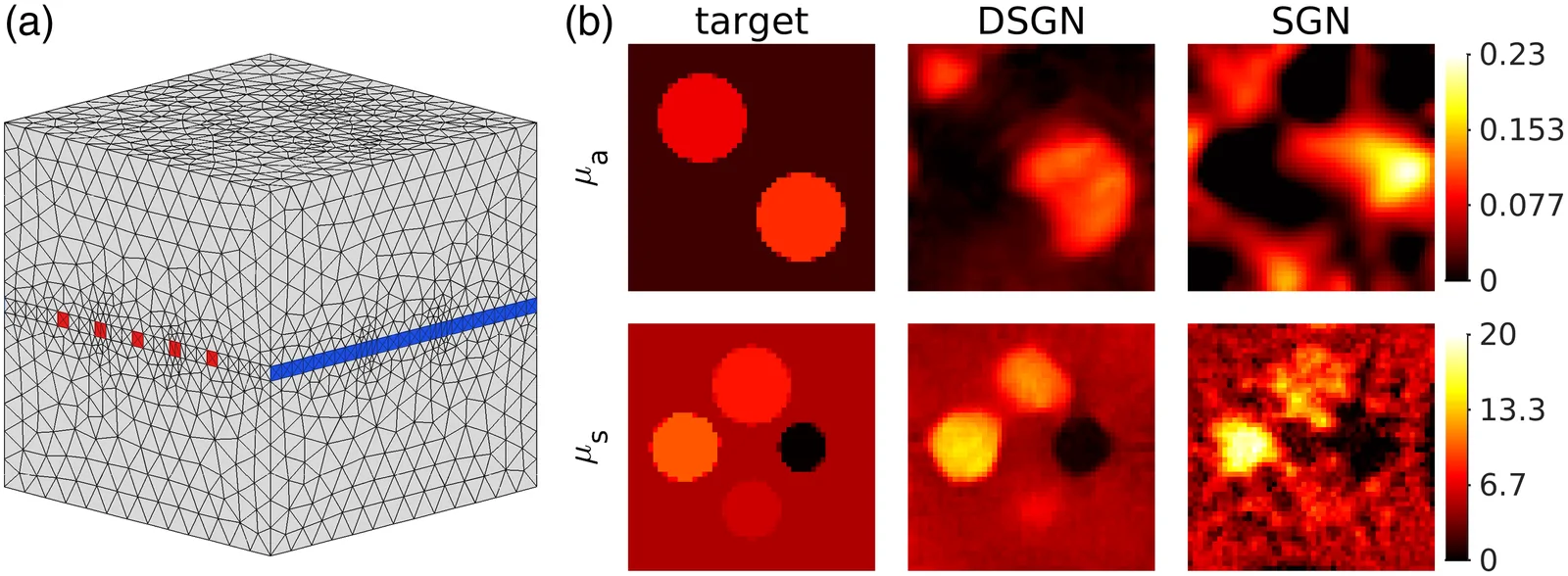
(a) 3D simulation domain with illustration of source (red) and detector (blue) positions on one side of the cube. (b) 2D reconstructions of absorption $\mu_{a}\;({\mathrm{mm}}^{-1})$ (first row) and scattering $\mu_{s}\;({\mathrm{mm}}^{-1})$ coefficients (second row) reconstructed from 3D simulated data. Columns from left to right: cross-section of the true target parameters in the middle of the target (first column), reconstructions calculated using the deep stochastic Gauss–Newton (DSGN, second column) and stochastic Gauss–Newton (SGN, third column) methods.

Furthermore, generalization of the 2D trained model to 3D data was studied. For this, we simulated Monte Carlo data in a $5\times 5\times 5\;{\mathrm{mm}}^{3}$ domain. Sources and detectors were placed on the boundary of the cube on the middle layer following the source and detector setup of the 2D imaging geometry, i.e., 20 sources (five on each side of the cube) and 100 detectors (25 on each side of the cube) were used. The domain was discretized using 35,639 tetrahedral elements, shown in Fig. 5, visualizing also the locations of sources and detectors on one side of the cube. The data were simulated using a target consisting of two absorbing and four scattering cylinders with 5 mm heights. The cross sections of the true target parameters in the middle of the target are also visualized in Fig. 5. Data were simulated using $10^{9}$ photon packets for each source. Then, the data were pre-calibrated to match the source strength between 2D and 3D models using a difference of data between 2D and 3D simulations with constant optical properties.

The reconstructed absorption and scattering coefficients calculated using the DSGN approach and compared against the SGN approach are shown in Fig. 5. Furthermore, the relative errors, Eq. (16), and SSIMs of the absorption and scattering estimates were computed after interpolating the true 3D absorption and scattering parameters to the 2D reconstruction mesh. These are given in Table 4. As can be seen, reasonable reconstructions can be obtained when the DSGN method (trained in 2D) is applied to 3D data.

**Table 4:** Relative errors $E_{\mu_{a}}(\%)$ and $E_{\mu_{s}}(\%)$ and structural similarity indexes ${\mathrm{SSIM}}_{\mu_{a}}$ and ${\mathrm{SSIM}}_{\mu_{s}}$ of absorption and scattering estimates calculated using the deep stochastic Gauss–Newton (DSGN) and the stochastic Gauss–Newton (SGN) methods. Results from generalization studies: data simulated in 3D, different levels of additive noise (1% and 5%) used in simulating the data, and different number of photon packets ($10^{5}$ and $10^{6}$) used in simulating the data.

	$E_{\mu_{a}}$ (%)		${\mathrm{SSIM}}_{\mu_{a}}$		$E_{\mu_{s}}$ (%)		${\mathrm{SSIM}}_{\mu_{s}}$	
Data type	DSGN	SGN	DSGN	SGN	DSGN	SGN	DSGN	SGN
3D	79.6	164.8	0.65	0.23	21.4	41.9	0.30	0.13
1% noise	40.7	51.4	0.89	0.80	8.4	15.1	0.43	0.25
5% noise	53.1	56.2	0.83	0.81	10.9	14.1	0.33	0.25
$10^{5}$ photon packets	66.0	209.3	0.77	−0.01	16.0	57.1	0.15	0.05
$10^{6}$ photon packets	48.7	81.9	0.86	0.58	9.2	27.4	0.37	0.15

#### 3.5.3 Generalization to different noise levels

Generalization of the DSGN method was also tested with data simulated using different levels of additive noise and different number of photon packets. Data simulated using $10^{8}$ photon packets and corrupted with additive noise of 1% and 5% and data simulated using $10^{5}$ and $10^{6}$ photon packets and corrupted with additive noise of 0.5% were studied. Notice that in the noise model of the reconstruction algorithm [noise covariance matrix $\Gamma_{e}$, Eqs. (1) and (8)], the standard deviation of the noise was modeled corresponding to simulated additive noise levels.

**Fig. 6:**
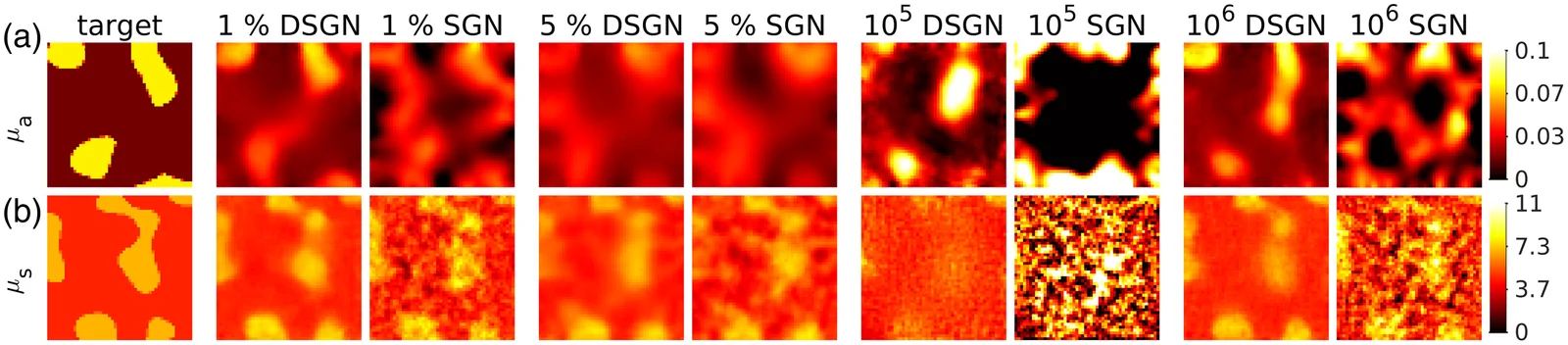
Reconstructed absorption coefficients $\mu_{a}\;({\mathrm{mm}}^{-1})$ (a) and scattering coefficient $\mu_{s}\;({\mathrm{mm}}^{-1})$ (b) of the target in the first row of Fig. 2. The true targets are shown in column 1. Columns 2 to 3 show deep stochastic Gauss–Newton (DSGN) and stochastic Gauss–Newton (SGN) reconstructions with 1% noise in simulated data, and columns 4 to 5 show reconstructions with 5% noise in simulated data. Columns 6 to 7 show DSGN and SGN reconstructions with simulated data using $10^{5}$ photon packets, and columns 8 to 9 show reconstructions with simulated data using $10^{6}$ photon packets.

The reconstructed absorption and scattering coefficients calculated using the DSGN approach and compared against the SGN approach are shown in Fig. 6. Furthermore, the relative errors, Eq. (16), and SSIMs of the absorption and scattering estimates are given in Table 4. As can be seen, the DSGN can improve SGN reconstructions both in the presence of higher additive noise and higher Monte Carlo noise. However, it does not generalize well if the noise statistics differ significantly from the statistics of the trained model and/or noise model of the image reconstruction algorithm, which is the case, for example, when data were simulated using $10^{5}$ photon packets.

#### 3.5.4 Comparison to deep-learned post-processing

**Fig. 7:**
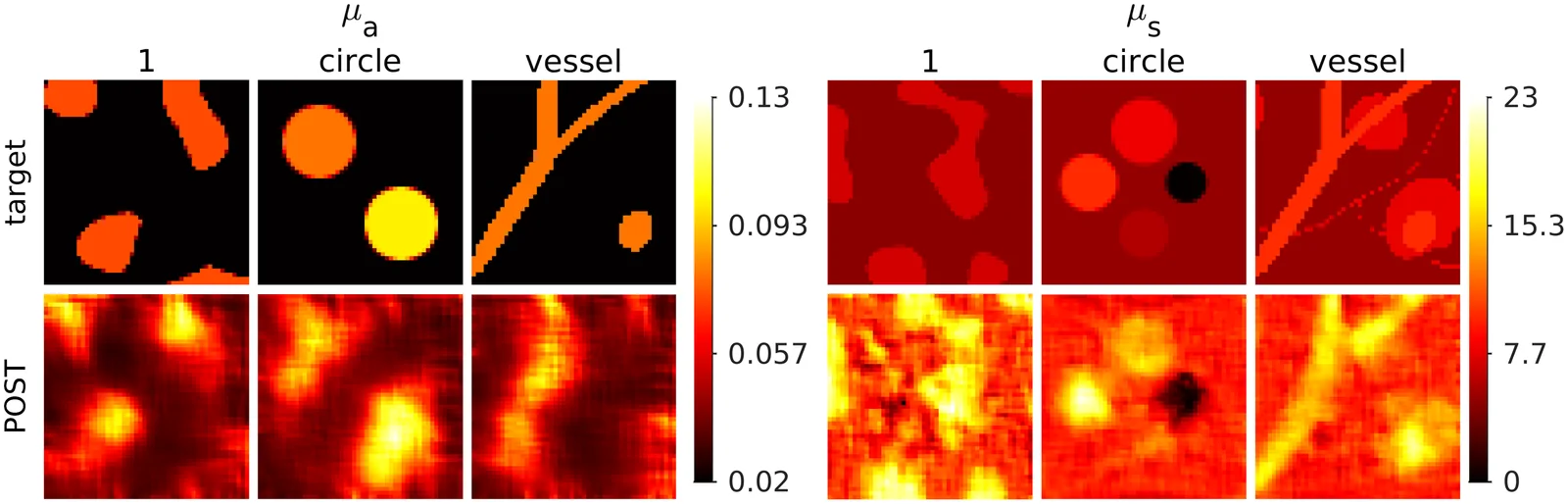
Reconstructed absorption coefficients $\mu_{a}\;({\mathrm{mm}}^{-1})$ (columns 1 to 3) and scattering coefficient $\mu_{s}\;({\mathrm{mm}}^{-1})$ (columns 4 to 6) of the target in the first row of Fig. 2, and the two out of distribution cases, circle and vessel. The true targets are shown in row 1 and the post-processing reconstructions (POST) in row 2.

We also compared the approach to a deep-learned post-processing methodology presented in Ref. 63. For the post-processing approach, the same network architecture that was used in Ref. 63 was applied to post-process the SGN absorption and scattering estimates obtained using $10^{6}$ photon packets. The post-processing networks for absorption and scattering were trained using the same 800 target samples (pair of absorption and scattering images) that were used to train the DSGN and their corresponding noisy SGN estimates. Examples of the post-processed absorption and scattering reconstructions from three targets (one in-distribution and two out-of-distribution) are shown in Fig. 7. The relative errors of these estimates were $E_{\mu_{a}}=78.9,65.4,78.8\%$, and $E_{\mu_{s}}=89.8,88.5,86.6\%$, respectively, and the structural similarity indices were ${\mathrm{SSIM}}_{\mu_{a}}=0.69,0.73,0.70$ and ${\mathrm{SSIM}}_{\mu_{s}}=0.22,0.18,0.26$, respectively. Comparing the reconstructions in Fig. 7 to the DSGN reconstructions in Figs. 2 and 4 and errors in Tables 2 and 3, we can see that the post-processed estimates show higher errors, especially for the out-of-distribution cases, where the scattering estimates show unrealistically high contrast.

## 4 Discussion and Conclusions

In this work, a model-based iterative deep learning approach was proposed for image reconstruction of absorption and scattering in OT, in the presence of stochastic noise due to using the Monte Carlo method for light transport as the forward model. In the approach, the search direction of a Gauss–Newton algorithm is replaced with a learned function on each iteration. The learned function is based on CNNs that are trained using a set of absorption and scattering images, and Gauss–Newton search directions that were calculated with a low number of photon packets. The performance of the approach was studied with simulations and compared against a conventional SGN algorithm and a deep-learned post-processing approach.

The results show that the proposed DSGN algorithm can be used to compensate for the stochastic noise that is due to using a low number of photon packets. As shown in Fig. 2, DSGN provides smoother images with better contrast and localization of the inclusions when compared with the SGN reconstructions using the same low number of photon packets ($10^{6}$). Furthermore, the DSGN provides reconstructions with better quality, lower mean relative error values, and higher mean structural similarity index values than the SGN method (Table 2). Furthermore, it was shown that the DSGN provides comparable reconstructions to SGN using a high number of photon packets ($10^{9}$), with similar relative error and structural similarity index levels. It should be noted though that the relative errors for all absorption and scattering estimates are relatively high. Thus, although OT can be used to reconstruct relatively low contrast targets and DSGN clearly improves quality of the reconstructions, further research is required for improving the quantitative accuracy of the methodology.

The generalization of the DSGN method was evaluated using two test targets that were outside the training set (Figs. 3 and 4), using 3D simulated data (Fig. 5), and using data simulated with different noise levels (Fig. 6). The results show that the method generalizes well to out-of-distribution targets and can be utilized in reconstructing various absorption and scattering distributions. The 2D DSGN reconstructions using the calibrated 3D data show a good level of generalization of the learned model to 3D when sources and detectors are located on a 2D layer and inclusions cross that layer. Regardless, the use of such 2D approximation model is not suitable in most of the practical applications of OT, and thus, future work includes, for example, extension of the methodology to full 3D. This will be computationally more expensive because it has been shown, for example, that the accuracy of Monte Carlo simulation depends on the discretization.^26^ In addition, 3D approach could be enhanced by machine learning approaches for model reduction, e.g., Ref. 43. Generalization of the methodology to different data with noise levels was also studied. It was noticed that the methodology generalizes well for different levels of additive and stochastic noise, but it does not generalize if noise statistics differ significantly from the statistics of the trained model and/or the noise model of the image reconstruction algorithm. Overall, the generalization could be improved by careful selection of the size and style of the training data, as well as further optimization of the training parameters, such as the batch size, number of epochs, and learning rate.

In all reconstructions, the noise in the scattering reconstructions was significantly higher than in the absorption reconstructions. We believe that this higher noise could be related to using the perturbation approximation of the Monte Carlo method^27^^,^^28^^,^^51^ in the evaluation of the Jacobian for scattering. An alternative approach would be to construct adjoint Jacobians using forward and adjoint photon density fields.^30^ However, this would require more research to compare these strategies and possible other methodologies for evaluation of Jacobians, as well as development of methodologies for compensating for stochastic noise.

The computation time of one iteration of the DSGN algorithm with $10^{6}$ photon packets was 28 s, whereas the SGN with $10^{9}$ photon packets took 4,547 s. On the other hand, convergence of the DSGN is slightly slower, i.e., average 15 iterations, than of the reference SGN method, i.e., average eight iterations. Thus, the average overall improvement in computation time, when iteration is stopped at convergence, is from 36,376 to 420 s, which means that the DSGN is approximately 85 times faster than the reference SGN with $10^{9}$ photon packets. We also note that training of the DSGN method is time-consuming, but it can be done offline, and after that, the trained model can be applied efficiently.

To conclude, it was shown that machine learning can be used to compensate for the uncertainties in the Gauss–Newton search direction due to stochastic Monte Carlo noise in OT. Furthermore, the methodology enables reconstructing absorption and scattering with a good quality using a significantly lower number of photon packets than a conventional stochastic Gauss–Newton algorithm.

## Data Availability

The data presented in this article are publicly available in the Monte Carlo Simulated Frequency Domain Optical Tomography Dataset at https://doi.org/10.5281/zenodo.19731513.
